# Transcription Factors in Cancer

**DOI:** 10.3390/ijms23084434

**Published:** 2022-04-18

**Authors:** Tomasz Wilanowski, Sebastian Dworkin

**Affiliations:** 1 Institute of Genetics and Biotechnology, Faculty of Biology, University of Warsaw, 1 Miecznikowa St., 02-096 Warsaw, Poland; t.wilanowski@biol.uw.edu.pl; 2 Department of Physiology, Anatomy and Microbiology, School of Life Sciences, La Trobe University, Bundoora, VIC 3086, Australia

This Special Issue comprises three original studies and five review articles. These publications highlight various aspects regarding the involvement of transcription factors in cancer. Two of the experimental papers address the functions of particular transcription factors in specific cancer types: SRY-box transcription factor 9 (SOX9) in chondrosarcoma and Yin Yang 1 (YY1) in B-cell non-Hodgkin lymphomas (B-NHLs) [1,2]. In these papers, both SOX9 and YY1 are discussed as potential drug targets because they both display tumor-promoting functions. In addition, both SOX9 and YY1 are relevant for the response of cancer cells to therapy. SOX9 is involved in many cancers, where it acts as a proto-oncogene or tumor suppressor, depending on the cancer type [3]. However, prior to this study, little was known about its role in chondrosarcoma. Stöckl et al. [1] discovered that the expression of SOX9 was increased in human chondrosarcoma biopsies, compared to normal cartilage, but it was decreased at later stages of the disease, when tumors became dedifferentiated. SOX9 promotes cell proliferation, clonogenicity and migration, and suppresses adhesion capacity, apoptosis and polyploidy formation, consistent with its postulated oncogenic role. Interestingly, SOX9 displays a very different influence on various anti-cancer therapies. It reduces the sensitivity of tumorigenic cells to doxorubicin treatment, does not affect their responses to cisplatin, and increases the efficiency of treatment of the T-VEC oncolytic virus. Clearly, these therapies stimulate different molecular pathways, which are discussed in detail in [1].

The article by Vivarelli et al. [2] focuses on the role of YY1 in reversing drug resistance in B-NHLs, as well as on regulating the expression of survivin, also known as a baculoviral inhibitor of apoptosis repeat-containing 5 (BIRC5) by YY1. YY1 is a dual-function transcription factor that can regulate the transcriptional activation and repression of many genes. Similar to SOX9, it can act as a proto-oncogene or tumor suppressor, depending on the cancer type [4]. In B-NHLs, YY1 acts as a tumor promoter, and this function is accomplished, at least in part, by its positive regulation of survivin expression [2]. The silencing of YY1 sensitizes B-NHL cell lines to doxorubicin and vincristine, which is indicative of its important response to therapy [2].

The third experimental publication in this Special Issue is concerned with the Raf kinase inhibitory protein (RKIP), also known as a phosphatidylethanolamine-binding protein 1 (PEBP1), and metadherin (MTDH) [5]. Technically speaking, RKIP and MTDH are not transcription factors, as they lack DNA-binding domains. However, we decided to include this article in the Special Issue, as MTDH can act as a cofactor for bona fide transcription factors, and thus associate with promoters of its target genes and regulate their expression [6]. MTDH, also known as LYRIC or the astrocyte-elevated gene-1 protein (AEG-1), has multiple roles in growth and development of various tumors, where it promotes all the hallmarks of cancer [7]. RKIP is a kinase inhibitor that regulates many signaling pathways, and in cancers, it functions as a tumor suppressor [8]. Indeed, its importance is so well-recognized that a Special Issue of *Cancers* was devoted entirely to this topic: *RKIP: A Pivotal Gene Product in the Pathogenesis of Cancer* [9]. In this Special Issue, Lai et al. [5] investigated the repression of RKIP by MTDH in breast and endometrial cancer cell lines. The regulation of RKIP expression was poorly understood previously; therefore, it is a significant finding that MTDH associates with the *RKIP* promoter and negatively regulates the expression of the *RKIP* gene [5].

The remaining five publications in this Special Issue are review articles. Krüppel-associated box zinc finger proteins (KRAB-ZFPs) constitute a family of transcription factors that primarily perform co-repressor functions in mammalian cells. Sobocinska et al. [10] explained the molecular mechanisms responsible for the functioning of KRAB-ZFPs and discuss in detail the involvement of nineteen members of this family in cancer. Their functions range from tumor suppressors to tumor promoters. There are also KRAB-ZFP factors that can perform either of the above roles, depending on the context. Particularly interesting are those members of this family that are implicated in response to chemotherapy [10]. The Ikaros zinc finger (IKZF) is another family of transcription factors reviewed in this Special Issue [11]. These factors are important for lymphocyte development and differentiation, and they also act as oncogenes in B cell malignancies, including multiple myeloma (MM). Cippitelli et al. [11] analyzed the significance of two members of the IKZF family, Aiolos and Ikaros, for the anti-MM activity of immunomodulatory drugs, such as thalidomide, lenalidomide and pomalidomide. This review is of particular interest regarding the response of tumorigenic cells to therapy.

Krüppel-like factor 4 (KLF4) acts as a tumor suppressor in many cancer types, but its functions and regulation are very complex. Taracha-Wisniewska et al. [12] discussed the diverse and multi-layered mechanisms of regulation of KLF4 activity. They also analyzed molecular mechanisms responsible for KLF4 involvement in various cancer types and warned against jumping to premature conclusions during investigations of this and other transcription factors associated with cancer development [12]. NF-E2-related factor 2 (NRF2) is a basic leucine zipper transcription factor primarily involved in the regulation of redox homeostasis. Its activity is regulated by its interaction with the Kelch-like ECH-associated protein 1 (KEAP1). The activation of NRF2 triggers molecular pathways that promote the development of hepatocellular carcinoma (HCC). Haque et al. [13] analyzed mutations in the *NRF2* and *KEAP1* genes that induced HCC development and discussed their relevant molecular mechanisms.

Endoplasmic reticulum (ER) stress leads to the accumulation of misfolded proteins and triggers unfolded protein responses (UPRs) [14]. This mechanism is very important in the liver, which is particularly susceptible to ER stress due to its protein synthesis and detoxification functions. Wei and Fang [14] analyzed its role in the pathogenesis of HCC. As the ER stress and UPR signaling pathway involves multiple transcription factors, we found this article to be very fitting for this Special Issue. Multiple molecular mechanisms are involved here, many of which are not completely understood [14].

In summary, transcription factors constitute the largest functional class of proteins in all living organisms. They regulate the expression of all genes and are located at critical points in signaling pathways. Their activity is altered in numerous cancer types. Many of them act as essential tumor suppressors, while others are key oncogenic drivers and are considered to be very promising therapeutic targets [15,16]. For these reasons we believe that the topic of this Special Issue is timely and of great interest to the wider scientific community.

## Data Availability

Not applicable.
